# Microstructural Characterization of Ball-Milled Biochar and Its Reinforcing Efficiency in Biobased Thermoplastic Polyurethane through Preferential Embedment in the Soft Segment

**DOI:** 10.1021/acssusresmgt.5c00225

**Published:** 2025-09-09

**Authors:** Kunal Manna, Chaoying Wan, Jaipal Gupta, James J. C. Busfield, Biqiong Chen, Ton Peijs

**Affiliations:** † Warwick Manufacturing Group (WMG), Centre for Polymers and Composites (CPC), 2707 University of Warwick, Coventry CV4 7AL, U.K.; ‡ School of Engineering and Materials Science, 4617 Queen Mary University of London, Mile End Road, London E1 4NS, U.K.; § School of Mechanical and Aerospace Engineering, Queen’s University Belfast, Stranmillis Road, Belfast BT9 5AH, U.K.

**Keywords:** biobased, thermoplastic elastomer, biochar, ball milling, characterization, mechanical properties, nanocomposites

## Abstract

In this study, we investigated the reinforcement effects of biochar on a bio-based thermoplastic polyurethane (bio-TPU). The particle size of the biochar was reduced and controlled by using a planetary ball milling process under varying milling conditions. The structure and morphology of ball-milled biochar (BBC) were thoroughly characterized using scanning electron microscopy (SEM), X-ray diffraction (XRD), X-ray photoelectron spectroscopy (XPS), Raman spectroscopy, and Brunauer–Emmett–Teller (BET) analysis. Bio-TPU/BBC composites were fabricated via melt compounding. The BBC was found to be preferentially localized within the soft segment (SS) phase of the TPU, as indicated by enhanced crystallization of the SS and a shift in its glass transition temperature (*T*_g_) to higher values. Two-dimensional small-angle X-ray scattering (2D SAXS) analysis revealed an increase in interdomain spacing from 11.22 to 12.09 nm with increasing BBC content, further supporting the preferential localization of BBC within the soft segments. This preferential reinforcement of the SS by BBC led to simultaneous improvements in both ultimate tensile strength (up to 35 MPa) and elongation-at-break (up to 780%) at a filler loading of 2.5 wt %. However, further increasing the BBC content to 10 wt % resulted in a decrease in elongation-at-break and toughness. Notably, the preferential embedment of BBC also contributed to a plateau stress of 8 MPa, addressing a known limitation in TPU design. Additionally, a 512% increase in Young’s modulus (YM) and a 26 °C improvement in the temperature corresponding to a 50% mass loss have been observed at 10 wt % BBC-filled bio-TPU composite, demonstrating a significant enhancement in the YM and thermal stability.

## Graphical abstract

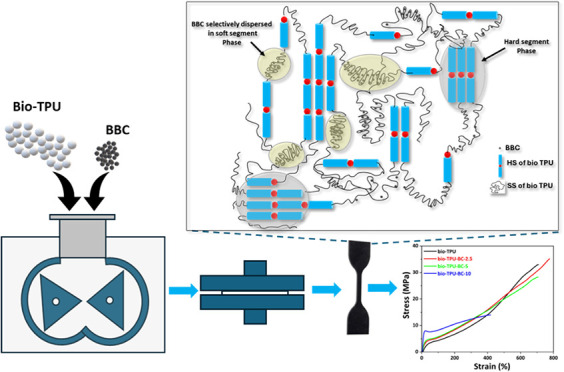

## Introduction

Mitigating global warming and climate change issues and increasing regulatory pressure from governments worldwide have accelerated research and development in bio-based and circular materials.^1,2^ Therefore, developing lightweight, renewable, and sustainable materials from bio-derived resources, recycled content, waste streams, or their combinations is essential to reduce carbon emissions and contribute to carbon sequestration.^3^ Consequently, research on developing renewable and eco-friendly biocomposites has recently dramatically increased. In this domain, polymer-based composites that use traditional carbonaceous and inorganic nanofillers have been the subject of many recent studies.^4–6^ In recent years, the shift from depleting petroleum-based resources to bio-renewable ones has sparked interest in the use of biomass as reinforcing fillers in polymers.^7^

In this context, biochar, being nontoxic, nonedible, and a carbon-negative material that can sequester carbon over a long period, aptly fits into the paradigm for new economic and social development, where it can be used for mitigating climate change and creating innovative sustainable biocomposites utilizing waste streams.^8,9^ Generally, biochar has a porous honeycomb structure with a high specific surface area, high hardness post-carbonization, and good thermal and chemical stability, making it an attractive filler for improving the thermo-mechanical properties of polymer composites.^10^ Biochar is also cheaper than almost all commercially available carbonaceous fillers and typically has a lower density. Biochar skeletal density ranges from 1.34 to 1.96 g·cm^–3^ and increases with pyrolysis temperature, and the biochar envelope density ranges from 0.25 to 0.60 g·cm^–3^.^11^ Given this, biochar can be qualified as a cost-effective, potential sustainable alternative to conventional fillers offering a wide processing window for blending with a variety of polymers to make cost-effective composite formulations with increased strength and stiffness in commodity or mid-performance applications.^12,13^ Though it is noteworthy to say that biochar is not expected to match the properties of high-performing carbonaceous nanofillers like CNTs or graphene, which are structurally different and used in fundamentally different contexts.

Despite these enormous possibilities, the major challenge with biochar lies in managing its broad range of sizes and shapes produced from different waste streams and biomass sources.^9^ However, it is well established that, compared to microparticles, nanoparticles generally offer better reinforcement in polymer composites in terms of high strength, thermal stability, and modulus.^7,14^ Additionally, it has also been reported that polymer composites prepared using reduced biochar particle size showed increased heat deflection temperature (HDT).^7^ A reduction of the size of biochar led to a reduction of interparticle distance, limiting the freedom of radius of gyration of the polymer macromolecules, especially in the amorphous phase. In consequence of this, higher temperatures were required to activate flow within the composite, leading to a higher HDT. Motivated by this, in the present work, we have adopted a planetary ball milling technique to prepare ball-milled biochar with significantly reduced particle size in comparison to raw biochar particles. The particles are reduced from a macro/microsize to a sub-micrometer or nanosize to work more effectively as a reinforcing filler and improve the interaction with different polymer matrices.

Up to now, the use of biochar as a filler has been explored on selective matrices like PP,^15,16^ PE,^17,10^ PET,^18^ PLA,^19^ PA,^20^ PVA,^21^ EVA,^22^ epoxy, etc.^23^ In most of these cases, an increase in the elastic moduli of the composites was achieved. In line with the transition from depleting petroleum-based resources to bio-renewable ones, the development of more sustainable polymeric or elastomeric derivatives with comparable mechanical and thermal properties has triggered an interest to counter the increasing global dependence on petroleum-based materials.^24^ Motivated by this, we have formulated sustainable biocomposites derived from biobased thermoplastic polyurethane (bio-TPU) reinforced with ball-milled biochar. Until now, the use of biochar in bio-TPU has rarely been reported.

TPU is a segmented copolymer that consists of alternate hard and soft segments along its backbone. Generally, soft segments (SSs) contain oligomeric polyols, etc., while the hard segments (HSs) consist of both isocyanates and chain extenders.^25,26^ TPU has a broad range of applications in specialty-molded parts, optical lenses, seals, gas separation membranes, shape-memory materials, and tribological fields such as transport belts, tires, rollers, and bushings due to its excellent abrasion resistance and mechanical properties.^27,28^ Recently introduced bio-based polyurethanes can play a significant role in promoting sustainability. These materials are part of the significant shift away from traditional petroleum-based feedstock for polymers toward renewable alternatives and include the use of a variety of different agricultural products and byproducts for the manufacture of TPUs.^29^ The problem with some of the TPUs is that they exhibit low stiffness and stress in the plateau region of their stress–strain curve.^29^

Mayakrishnan et al.^30^ prepared TPU/nanobiochar nanocomposite mulch films with 110% improvement in tensile strength at 10 wt % loading of nanobiochar using a fused deposition modelling technique. Lepak-Kuc et al.^31^ prepared TPU/biochar composites and investigated mechanical, thermal, and electrical properties. They achieved a 55% improvement in ultimate tensile stress (UTS) with a 4% reduction in elongation-at-break (EB) at 60 wt % biochar loading. Uram et al.^32^ prepared rigid polyurethane foam with different biochar loadings (3 to 20 wt %) and investigated their mechanical properties, thermal stability, and thermal conductivity. They observed with increasing biochar content a reduction in stiffness and compressive strengths at 10% strain. So far, all studies in the literature have focused on the preparation of TPU/biochar composites using non-biobased synthetic TPUs.^33^ Furthermore, the underlying reinforcing mechanism along with the location of the biochar in the TPU matrix, being in either the SS or HS, has not been discussed in great detail. Therefore, there is potential in developing bio-based TPU/biochar composites and investigating the reinforcing effects of ball-milled biochar.

This work explores the development of sustainable biocomposites derived from ball-milled biochar and a commercial biobased TPU elastomer like ESTANE ECO 12T80E. The novelty of this investigation lies in the fact that, to the best of our knowledge, this is one of the first studies to develop sustainable biocomposites derived from a commercially available biobased TPU matrix reinforced with different amounts (2.5 to 10 wt %) of a commercially available biochar postball milling without any other additive. These sustainable biocomposites offer a simultaneous increase in ultimate tensile strength (UTS) and elongation at break (EB) at an optimum loading of ball-milled biochar, which is a rare observation in biochar-filled systems. Furthermore, an increase in Young’s modulus (YM) in the biobased TPU has been achieved. Such improvement could be anticipated through preferential embedment of the ball-milled biochar in the soft segments of the bio-TPU. Consequently, high stiffness and stress in the plateau region have been achieved in these TPU-based composites.

## Experimental Section

### Materials

High-performance biobased thermoplastic polyurethane (bio-TPU) ESTANE ECO 12T80E with ∼43% biocontent was supplied by Lubrizol, USA. The property profile of this bio-TPU (density: 1.10 g/cm^3^) is comparable to a standard TPU of the same hardness (A/1:82 - Shore A/D), along with excellent mechanical properties and abrasion resistance. Biochar having a broad size distribution (carbon content: 88.53%, ash content: 8.5%, sieve >2 mm: 92.88%, H.L. weight or grain density: 160.3 kg/m^3^) was kindly supplied by Joss Elastomers and Chemicals, Netherlands. This biochar was produced by the pyrolysis of sawmill waste wood at 450–500 °C.

### Methods

#### Standardized Ball Milling of Biochar

Ball milling was performed in a planetary ball mill (PM100; Retsch Corporation) using an 80 mL stainless steel jar and stainless steel balls of 5 mm in diameter. To standardize the ball milling time to reduce the size of the biochar to below 1 μm, the remaining parameters including rotational speed (450 rpm), ball-to-sample mass ratio (10:1), interval time (15 min), and breaking time (5 min) were kept constant. The time intervals with direction reversal were set to 15 min ON and 5 min OFF as the breaking time to prevent overheating of the biochar and balls. To investigate the morphological evolution of the as-received biochar after ball milling, scanning electron microscopy (SEM) analysis was performed on the ball-milled biochar by removing it from the jar after a break time of 5 min and after each interval of 15 min. Finally, the ball milling time was standardized up to 3 h to achieve an average particle size of less than 1 μm, keeping all of the other parameters the same. This ball-milled biochar was designated as ball-milled biochar (BBC), and the as-received raw biochar was designated as the raw biochar (RBC).

#### Processing of Bio-TPU/BBC Biocomposites

Bio-TPU granules were first dried overnight in a vacuum oven at 80 °C. A Thermo HAAKE internal mixer (model HAAKE PolyLab OS, RheoDrive 7) was used for melt-mixing at a rotor speed of 50 rpm and a mixing temperature of 200 °C. To adopt an efficient way to disperse BBC in the polymer matrix, bio-TPU was allowed to initially melt for 5 min at 200 °C, followed by the addition of the desired amount of BBC particles, while mixing continued for another 5 min with a total mixing time of 10 min. The mass of bio-TPU granules in the internal batch mixer was fixed to 50 g, followed by the addition of a varying amount of BBC (2.5, 5, and 10 wt %, *x*) to prepare biocomposites designated as bio-TPU-BC-*x*. Thin (1 mm) films were obtained by hot pressing using a COLLIN hot press (temperature of 200 °C, pressure of 10 MPa, time of 9 min) followed by cooling under pressure to room temperature. Further, an ISO 5893 standard A type 2 dumbbell test specimen was punched out for tensile testing.

### Characterization Techniques

The as-obtained raw biochar (RBC) and the ball-milled biochar (BBC) were characterized using SEM, powder X-ray diffraction (PXRD), X-ray photoelectron spectroscopy (XPS), X-ray-induced C-KLL Auger spectra, RAMAN spectroscopy, and Brunauer–Emmett–Teller (BET) specific surface area analysis to investigate the effect of ball milling. Further, the fabricated Bio-TPU/BBC biocomposites were characterized by powder XRD, wide-angle X-ray scattering (WAXS), mechanical property analysis, hardness testing, differential scanning calorimetry (DSC), dynamic mechanical analysis (DMA), two-dimensional small-angle X-ray scattering (2D SAXS), SEM, and thermogravimetric analysis (TGA). All the instrumental details and procedures of the adopted characterization techniques are described in detail in the supporting information (SI–I) of the article.

## Results and Discussion

The morphological changes in the biochar during ball milling were captured through SEM images. The gradual size reduction of the RBC particles is observed in Figure S1a–c under supporting information after 15, 30, and 60 min of ball milling, respectively. The RBC particles have a broad size distribution of 5–500 μm, as shown in Figure 1a. A SEM image of BBC particles after a standardized ball milling time of 3 h with an average sub-micrometer particle size of around 500 nm is shown in Figure 1b. The reduction of biochar particle size to nanometric levels could be ascribed to the breakdown of the biochar particles due to the increased impact, shear, frictional forces, and temperature exerted on the particles during ball milling.^34^ Such a nanometric level of particle sizes is expected to facilitate better reinforcing ability even in the agglomerated form on account of van der Waals attractive forces in the system.^35^ Hence, this nanosized biochar is likely to offer improved mechanical properties and thermal stability in the composites when combined with bio-TPU.

**Figure 1:**
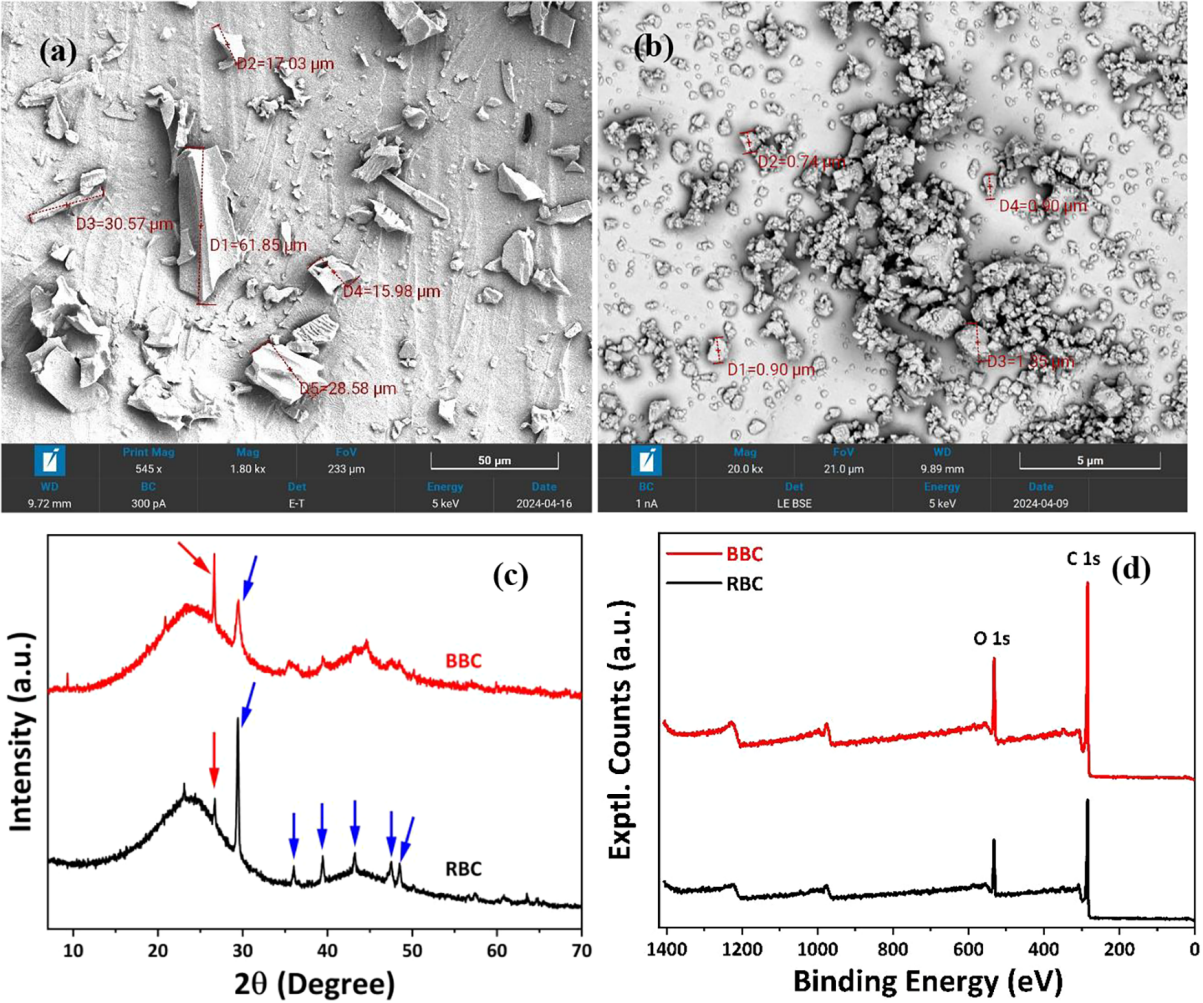
SEM image of (a) RBC and (b) BBC after 3h ball milling. (c) Powder XRD and (d) XPS spectra of RBC and BBC.

The XRD patterns of the RBC and BBC are shown in Figure 1c. XRD data have been analyzed using the semiquant method that compares known XRD patterns to the measured data to determine the phases present and estimate the fraction. The crystalline material was identified as calcium carbonate (CaCO_3_) and quartz. The positions of the diffraction peaks in Figure 1c are denoted by blue arrows for CaCO_3_ and a red arrow for quartz. The reduction in peak intensity and increase in peak width for the CaCO_3_ peaks in BBC suggest that the ball milling process has reduced the crystallite size. The quartz peak at 2θ ∼27° has an increased peak width but has increased in intensity in BBC compared to RBC. This could be due to the reduction in crystallite size and any preferred orientation of the quartz crystallites.^36^ As per the semiquant analysis, RBC consists of 85% CaCO_3_ and 15% quartz, and BBC contains 37% CaCO_3_ and 63% quartz. Such a change in the estimated % can be attributed to a transition of sp^2^ to sp^3^ as C in standard CaCO_3_ is sp^2^ hybridized, while Si in quartz is sp^3^ hybridized. This was later corroborated by XPS. From XPS analysis, it is seen that carbon and oxygen are prominent in both RBC and BBC, and the peak intensity of both C 1s and O 1s has increased after ball milling as observed from the survey scan spectra in Figure 1d. The quantitative analysis report of the elemental composition of RBC and BBC as obtained through XPS analysis is presented in Table 1 where a minor presence of K and N has also been detected. For an in-depth understanding of the effect of ball milling on the bonding environment, both the C 1s and O 1s peaks of RBC and BBC were deconvoluted.

**Table 1:** XPS Elemental Composition of RBC and BBC (Atomic %)

elemental composition (atomic %)				
samples	C	K	O	N
RBC	81.2	0.6	17.3	1
BBC	81.3	0.3	18.4	0

The relative percentage of the different bonding regions obtained after deconvolution of C 1s and O 1s is reported in Table S1 under supporting information. It is observed in Figure 2a,b that the sp^2^ C–C peak intensity has decreased and the sp^3^ C–C/C–H peak intensity has increased after ball milling. Similarly, from the O 1s deconvoluted spectra in Figure 2c,d, it can be inferred that the sp^2^ >C=O and moisture (H_2_O) peak intensity has reduced after ball milling because of the reduction in sp^2^-rich domain and absorption of moisture due to heat generated during the milling process. To further quantify the sp^2^ to sp^3^ transitions and obtain additional information regarding the arrangement of C atoms on the surface, a more surface-sensitive C-KLL spectra analysis was performed on RBC and BBC.

**Figure 2:**
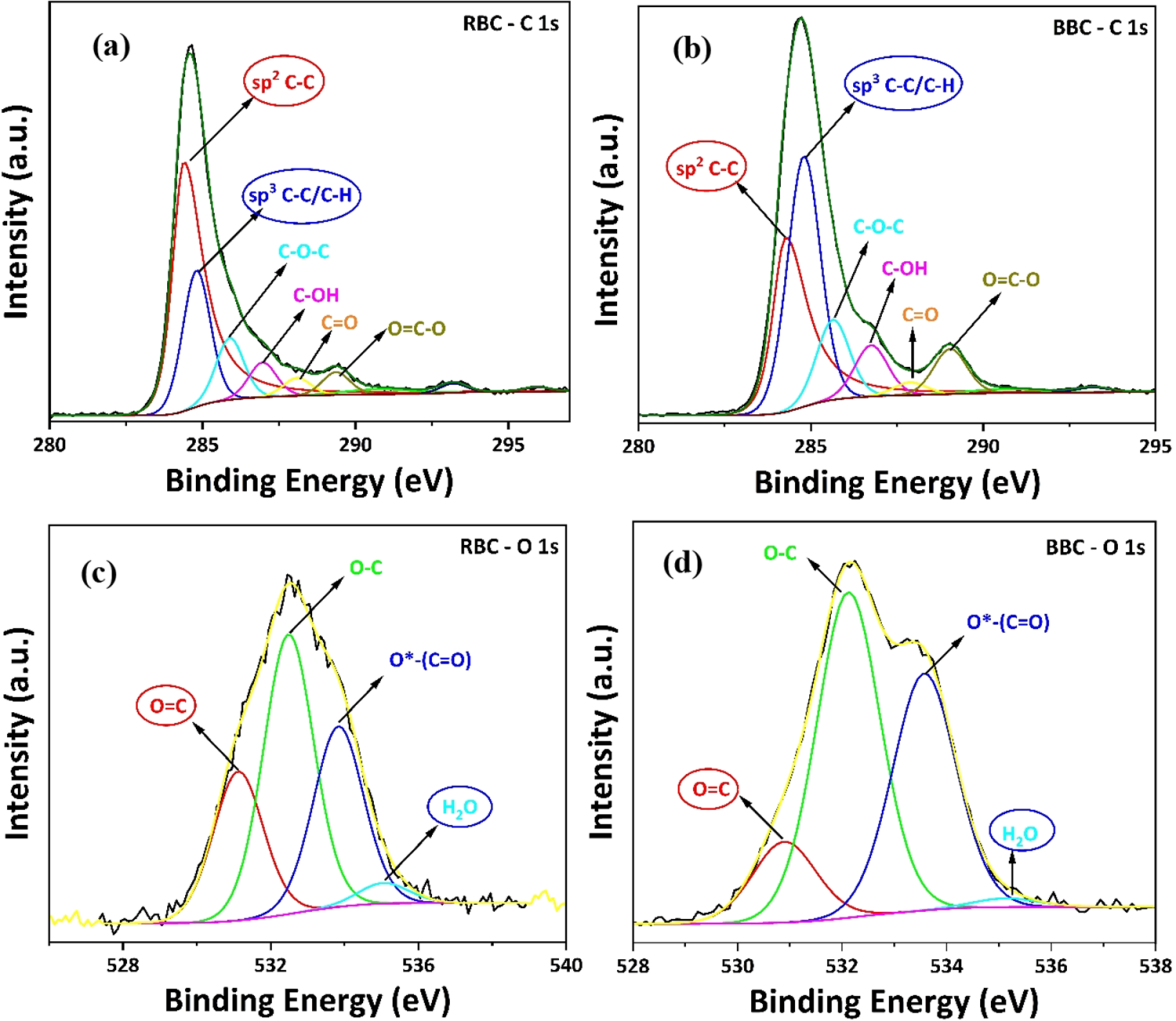
Deconvoluted XPS spectra of (a) RBC-C 1s, (b) BBC-C 1s, (c) RBC-O 1s, and (d) BBC-O 1s.

Figure S2a under supporting information shows the C-KLL spectra of RBC and BBC, and the spectral feature changes according to the arrangement of C atoms in the surface under investigation.^37,38^ The sp^2^ to sp^3^ transitions can be quantified from the D-parameter elucidated from the energy difference between the maximum and minimum of the differential curves as depicted in Figure S2b. Typical values of the D-parameter range from 11 to 13 eV for sp^3^-like materials and 21 to 23 eV for sp^2^-like materials.^39^ The calculated *D*-parameters for RBC and BBC are 15.9 and 15.6 eV, respectively, which sit between sp^3^- and sp^2^-rich materials. The shift of the *D*-parameter to a lower value of BBC further confirms the sp^2^ to sp^3^ transition and a more sp^3^-rich domain on the surface after ball milling.

To further investigate the degree of defects, disorder, or lattice distortion after ball milling of biochar, Raman spectroscopic analysis was carried out on RBC and BBC. Details of the analysis and a full Raman spectrum (200–3500 cm^–1^) of BBC are given in Figure S3a of the supporting information. Insignificant changes in the intensity ratio of D and G bands have been noted in Figure S3b, thereby confirming that ball milling is a nondestructive method for inducing nanoscale changes in biochar microstructure.^18,40,41^ For further information, the Raman spectra of RBC and BBC were deconvoluted to give insight into their D, D′, D″, and G bands as depicted in Figure S3c,d in the supporting information, respectively, and a signature for sp^2^ to sp^3^ transition.

The evolution of volatiles during the high-temperature carbonization process of biochar leaves a porous structure.^18^ Therefore, detailed BET analysis including the specific surface area, average pore size, and pore volume was done on RBC and BBC, and the corresponding findings are summarized in Table 2. As per the results, BBC particles possess a much higher surface area and pore volume compared to RBC. The N_2_ adsorption–desorption isotherm in a partial pressure range of 0.03–0.99 is presented in Figure 3a.

**Figure 3:**
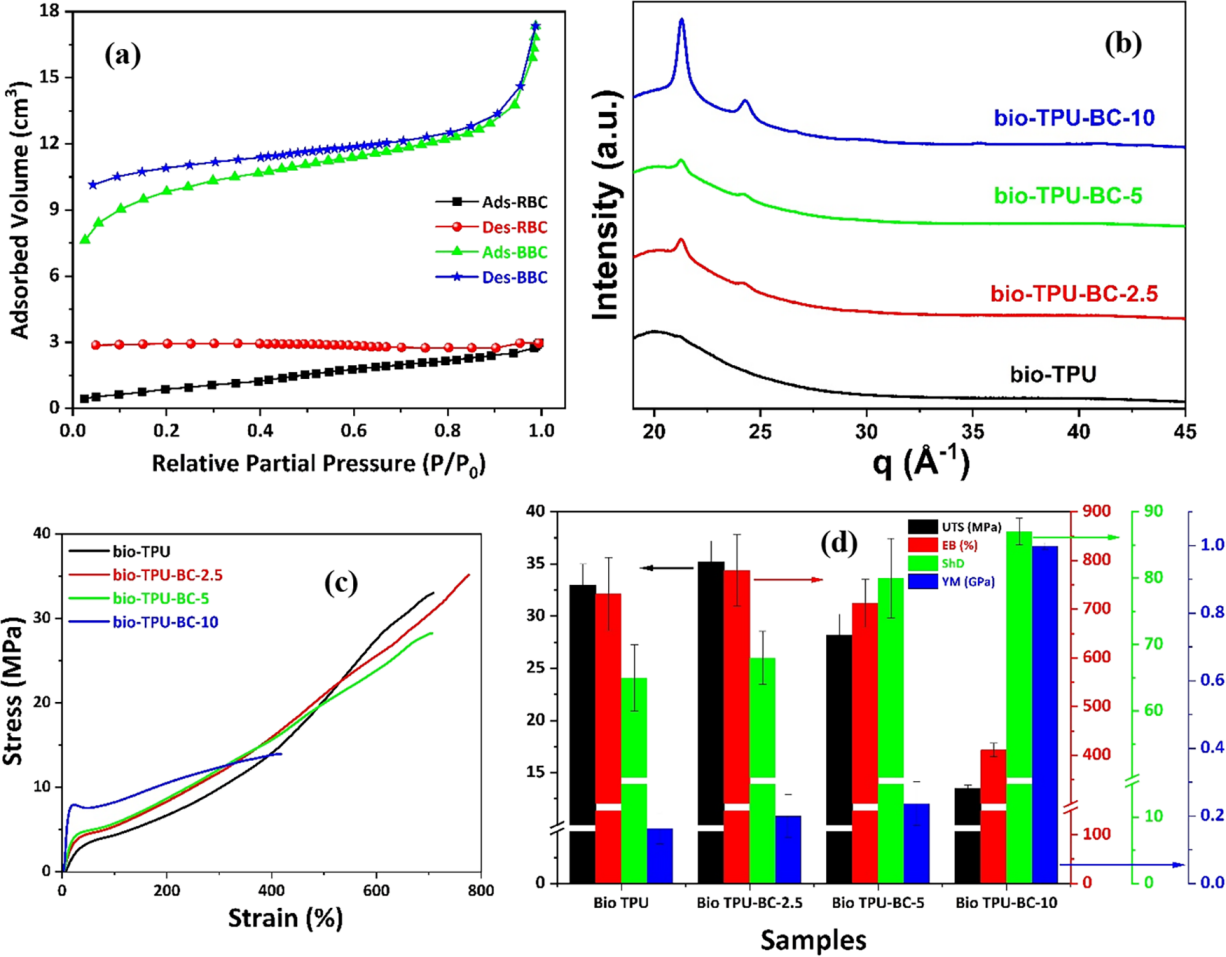
(a) N_2_ ads-des isotherm of RBC and BBC. (b) WAXS pattern of bio-TPU/BBC biocomposites. (c) Stress vs Strain plot. (d) UTS, EB, ShD, and YM value of bio-TPU/BBC biocomposites.

**Table 2:** Comparison of BET Analysis Results of RBC and BBC

sample	BET surface area (m^2^/g)	pore volume (cm^3^/g)	average pore size (nm)
RBC	40	0.043	3.3
BBC	299	0.120	3.5

The most obvious finding is that the hysteresis loop does not close for both RBC and BBC even at low *P*/*P*_0_. Similar findings have been previously reported in the literature.^42–44^ This implies that BBC has a greater residual adsorption capacity compared to RBC with more developed micropores that are not easy to desorb. This can be further supported by the plot of cumulative pore volume versus pore diameter in Figure S4a in the supporting information. The RBC had fewer pores below 50 nm with an average pore size of 3.3 nm. In contrast, the BBC particles possess slightly more pores below 50 nm with an average pore size of 3.5 nm. This leads to a higher cumulative pore volume for BBC (0.12 cm^3^/g) compared to RBC (0.043 cm^3^/g) in the entire pore diameter range of 0–300 nm. Consequently, higher pore surface area is reflected in Figure S4b (in supporting information) in the case of BBC contributed by its micro as well as mesopores ranging from 2 to 50 nm.^45^ The BJH (Barrett–Joyner–Halenda) pore size distribution of RBC and BBC is shown in Figure S4c in the supporting information. For a pore diameter below 20 nm, d*V*/d*D* increases sharply with decreasing pore size for both RBC and BBC with the higher growth rate being observed for BBC, highlighting the pronounced effect of deformation and metamorphic changes in biochar after ball milling.^42^

Powder XRD patterns of bio-TPU/BBC biocomposites are shown in Figure S4d under the supporting information. For pristine bio-TPU, relatively broader peaks are observed at 2θ ∼20.5°, which can be assigned to hard domain crystalline phases.^46^ After BBC particles were incorporated, two relatively sharp peaks emerged in the composites. One main peak at 2θ ∼21.2° and another less intense shoulder peak at 2θ ∼24.2° are observed. Interestingly, neither of these peak positions matches the peaks of powder XRD of BBC as shown in Figure 1c. Therefore, these newly emerged peaks could be attributed to the induced crystallinity in bio-TPU after the incorporation of BBC particles. To verify these unusual findings, we performed wide-angle X-ray scattering (WAXS) analysis of the bio-TPU/BBC composites, which showed that both peaks are visible in the same position (Figure 3b). Moreover, the intensities of these two peaks have gradually increased with the loading of BBC as evidenced by both XRD and WAXS patterns. As the degree of crystallinity influences the mechanical properties of semicrystalline polymer-based composites, the tensile properties of the bio-TPU/BBC biocomposites were assessed.

Figure 3c shows the representative stress–strain curves of bio-TPU and bio-TPU/BBC composites of various BBC loading (2.5–10 wt %), which can be divided into three regions.^4,26,47^ In the low-strain region, the curves are almost linear with a slope equivalent to the Young’s modulus (YM). At higher strains, the curves flatten to give a small plateau region associated with the continued stretching of the SS and rotation and alignment of the HS.^26^ The plateau region is followed by a high-slope region associated with strain hardening (SH) and strain-induced crystallization (SIC). At high strain values, the samples eventually fractured.^26^ The general trade-off of a decrease in extensibility at higher loading (5 and 10 wt %) of BBC beyond 2.5 wt % has been observed. The maximum Young’s modulus was achieved in bio-TPU-BC-10 at the highest loading of BBC, sacrificing extensibility as well as UTS. BBC particles are found to be ∼500 nm, as earlier inferred from FESEM image (Figure 1b), while typical hard segment (HS) domains in TPU are 2–10 nm in diameter. This significant size mismatch makes it physically implausible for BBC to be embedded within or interact directly with HS domains. Instead, dispersion into the continuous soft segment (SS) matrix is far more likely. Therefore, it can be assumed that the BBC particles are preferentially embedded within the soft microdomains of the bio-TPU, thereby hindering the mobility of these segments, making them more difficult to stretch and align, resulting in a sharp decline in elongation at break (EB).^26^ Further, the decrease in UTS could be attributed to inefficiency in stress transfer and inhomogeneous dispersion of the BBC in the bio-TPU matrix. The observed increases in stiffness and stress in all three regions of the stress–strain curve, coupled with the sharp decrease in extensibility with increasing BBC content in the biocomposites can be ascribed to the preferential embedment of BBC within the SS of bio-TPU. Other than this, two important parameters for elastomeric composites are toughness and hardness. The toughness of the prepared bio-TPU/BBC bio-composites follows the order, bio-TPU-BC-2.5 > bio-TPU > bio-TPU-BC-5 > bio-TPU-10. Contrary to this, the Shore D hardness (ShD) showed the opposite trend as the ShD increases monotonically with BBC content. The UTS, EB, ShD, and YM values are all listed in Table 3 and collated in Figure 3d as a bar graph with multiple *y*-axis. It is evident that incorporating 2.5 wt % BBC into bio-TPU results in approximately 6 and 7% increases in UTS and EB, respectively, compared to neat bio-TPU. As per previous reports, the enhancement in UTS in TPU-based composites without compromising the elongation at break has been achieved by selective reinforcement of HS.^4,26,48^ To judge the potentiality of BBC as a reinforcing filler to boost the mechanical property of polymer-based composites, we have compared our findings with other conventional carbonaceous and inorganic fillers (like CB, graphite, CNT, and Silica)-based TPU systems from the literature. Dong et al.^49^ reported almost 15% improvement in UTS and 8% improvement in EB for TPU/CB composites at 8% loading of CB. In another work, Zhao et al.^50^ have reported 10% improvement in UTS at the cost of ∼68% reduction of fracture strain for the three-dimensional (3D) printed TPU/CB composites at 5% loading of CB. In another study, 3D-printed biocompatible TPU composites with three different types of conductive carbonaceous fillers (CNT, graphite, and CB) were prepared, and their properties were investigated in a comparative manner. When directly comparing the UTS values of neat TPU against TPU loaded with 10 wt % of CNT, graphite, or CB, the values reduced by 43, 35, and 90%, respectively. In the case of EB for the same comparison, the values reduced by 50, 19, and 97%, respectively.^51^

**Table 3:** Mechanical Properties and Shore D Hardness of Bio-TPU/BBC Biocomposites

samples	UTS (MPa)	EB (%)	YM (MPa)	ShD
bio-TPU	33.0 ± 2.02	731 ± 110	0.163 ± 0.045	65 ± 5
bio-TPU-BC-2.5	35.2 ± 2.32	780 ± 73	0.199 ± 0.064	68 ± 4
bio-TPU-BC-5	28.2 ± 2.06	712 ± 49	0.236 ± 0.065	80 ± 6
bio-TPU-BC-10	13.5 ± 0.95	411 ± 14	0.999 ± 0.100	87 ± 3

However, in our case, the % reduction of UTS and EB compared to that of neat bio TPU at 10 wt % loading of BBC is 60 and 44%, respectively. For TPU/fumed silica nanocomposite, the highest improvement in UTS (∼33%) has been reported by Saha et al. with a slight reduction in EB (∼1.5%) at 1 wt % loading of nanosilica.^52^ In this context, our present findings of a simultaneous increase in the levels of UTS and EB in the case of bio-TPU-BC-2.5 are interesting and require further in-depth analysis. Therefore, DSC analysis was done to reveal the underlying reinforcing mechanism of BBC and to check its preferential insertion in either the SS or the HS.

The DSC curves of the 2nd heating cycle of bio-TPU and its biocomposites showed a hump-like exothermic peak associated with SS crystallization around −10 °C in between the endothermic peaks of the glass transition (*T*_g_) around −45 to −30 °C and melting (*T*_m_) of the SS (Figure 4a). Such an exothermic SS crystallization peak in the heating cycle has previously been reported for TPU.^26,53–55^ The cooling curve is depicted in Figure 4b, and the thermal transitions are recorded in Table 4. Up to five main transitions were observed for the bio-TPU and the bio-TPU/BBC biocomposites, including *T*_g_ of SS, *T*_m_ of SS and HS, and the crystallization temperature (*T*_c_) of SS and HS. The significant increase in *T*_m_ and melting enthalpy (ΔH_m_) of SS after the addition of BBC suggests an increase in the nucleation of SS crystallinity in the presence of BBC.^22^ In contrast, the decrease in the *T*_m_ of HS reflects a less crystalline packing potential. This phenomenon can be explained by the preferential embedment of BBC within the SS of bio-TPU. Such prominent nucleating effect of BBC can be further intensified from the abrupt increase in *T*_c_ from −19 to +17 °C and the crystallization enthalpy (Δ*H*_c_) of SS coupled with an increased peak intensity at higher BBC loadings as observed in Figure 4b. However, an insignificant increase in *T*_c_ (HS) associated with the reduction in peak intensity at higher BBC loading implies that less energy is needed for the crystallization of HS, as inferred earlier.^48^ However, the presence of clear melting [*T*_m_ (HS) 147–164 °C] and crystallization transitions [*T*_c_ (HS) 73–79 °C] indicates that the HS domains are semicrystalline, not glassy. Therefore, these domains are dense and ordered, further supporting the notion that large BBC particles cannot enter or disrupt them directly. These findings further substantiate the preferential reinforcement of SS through predominant dispersion of BBC. As discussed earlier, the SS *T*_g_ values of bio-TPU and its biocomposites obtained from DSC fall in the range of around −45 to −30 °C; therefore, for the present case, capturing the glass transition more precisely seems to be difficult using DSC. Since *T*_g_ values are a signature of segmental motion of molecular chains of SS, DMA characterization of the bio-TPU/BBC composites was carried out at 1% strain amplitude to detect a potential shift of *T*_g_ and identify the preferential location of BBC in bio-TPU.^4^

**Figure 4:**
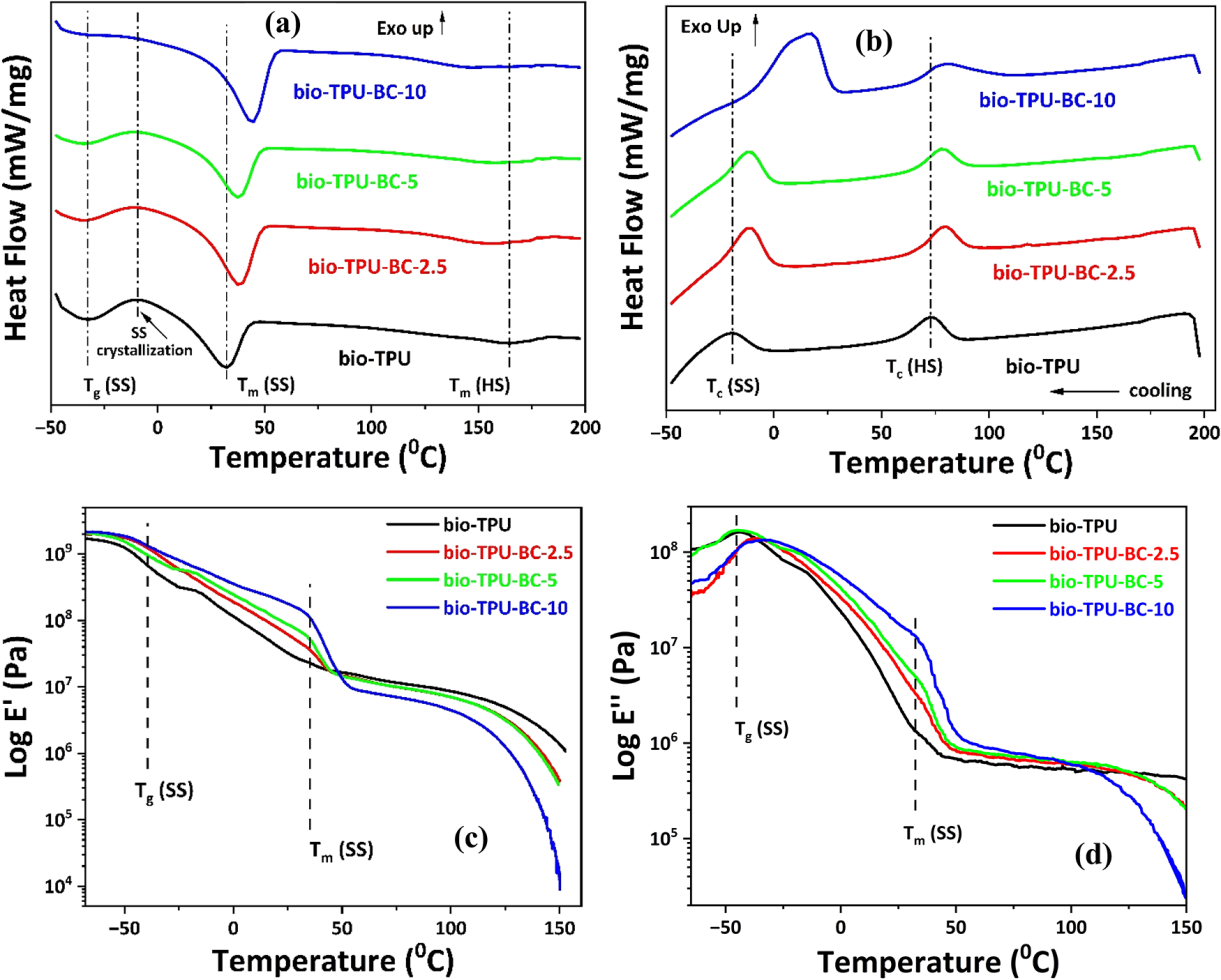
DSC curves of bio-TPU/BBC biocomposites’ (a) 2nd heating cycle showing an increase in *T*_m_ (SS) due to restricted segmental motion on account of preferential location of BBC and (b) cooling cycle, showing shift in *T*_c_ (SS) and *T*_c_ (HS), which also indicates lower segmental motion. Plot of (c) Storage moduli (*E*′) and (d) loss moduli (*E*″) vs temperature.

**Table 4:** Thermal Transition Values of Bio-TPU and Bio-TPU/BBC Biocomposites

samples	*T*_m_ (SS) (°C)	*T*_m_ (HS) (°C)	Δ*H*_m_ (SS) (J/g)	*T*_c_ (SS) (°C)	*T*_c_ (HS) (°C)	Δ*H*_c_ (SS) (J/g)
bio-TPU	32	164	–26.3	–19	73	13.5
bio-TPU-BC-2.5	38	156	–29.4	–11	80	19.2
bio-TPU-BC-5	37	158	–25.2	–12	78	16.6
bio-TPU-BC-10	44	147	–37.0	17	79	29.7

The temperature dependence of storage moduli (*E*′) and loss moduli (*E*″) for the bio-TPU and the BBC-filled biocomposites is shown in Figure 4c,d. The storage moduli values of bio-TPU/BBC biocomposites in the glassy state in the temperature range of −65 to −50 °C are slightly higher compared to neat bio-TPU due to their higher crystallinity as reflected earlier by the Δ*H*_c_ (SS) values in the DSC data (see Table 4).^56,57^ At higher temperatures, the materials show a decrease in *E*′ associated with the *T*_g_ as is evident in Figure 4c. After this decrease in *E*′, the biocomposites reach a plateau in the temperature range of −30 to +35 °C before the melting of SS. In this region, *E*′ values of the bio-TPU/BBC biocomposites increase with increasing BBC content from 2.5 to 10 wt %. Such increment in *E*′ could be ascribed to significant structural reinforcement of the SS phase of the bio-TPU through preferential embedment of BBC exhibiting higher *T*_m_ (SS) as revealed by DSC.^57^ Finally, at higher temperatures after the melting of SS, the materials reach a second plateau, where the opposite trend in the *E*′ values has been observed. In this temperature region, starting from 45 °C up to 110 °C, the *E*′ values started to decrease with increasing BBC content, while above 110 °C, the bio-TPU-BC-10 starts to flow. This suggests less or no interaction of BBC particles with the hard segments of the bio-TPU.

Analyzing Figure 4d, which shows the logarithmic values of loss moduli as a function of temperature, it can be concluded that the higher *E*″ values for neat bio-TPU and bio-TPU-BC-5 can be accounted for by the absence of BBC or the agglomeration of BBC, leading to reduced stiffness and UTS.^58^ However, lower *E*″ values in 2.5 and 10 wt % BBC composites imply reduced mobility and increased stiffness of the biocomposites in agreement with the highest UTS and YM for the corresponding biocomposites, respectively. The shift in *T*_g_ of the SS to higher temperatures for bio-TPU-BC-2.5 and bio-TPU-BC-10 is as per the above findings.

The maximum tan δ values in Figure 5a vary between 0.25 for neat bio-TPU and 0.12 for bio-TPU-BC-10. More specifically, *T*_g_ (SS) as obtained from the apex of the tan δ curves shows a shift to the right, demonstrating an increase in *T*_g_. The *T*_g_ calculated from the peak of tan δ increased from −24 °C for neat bio-TPU to −12 °C for bio-TPU-BC-2.5. This can again be explained by the preferential embedment of BBC in the SS phase of bio-TPU, which effectively restricts the mobility of polymer chains in the SS phase.^48^ Furthermore, the increase in *T*_g_ in bio-TPU-BC-10 up to −11 °C, together with peak broadening and the appearance of a 2nd peak at higher BBC loadings, indicates mild phase separation between SS and HS.^59^ All of these arguments, facts, and findings suggest that BBC has preferentially reinforced the SS phase of bio-TPU, leading to an increase in UTS as well as EB at an optimum filler loading of 2.5 wt %. This is a relatively rare and interesting observation in biochar-filled systems, and to further demonstrate such a preferential reinforcement, 2D-SAXS analysis was employed.

**Figure 5:**
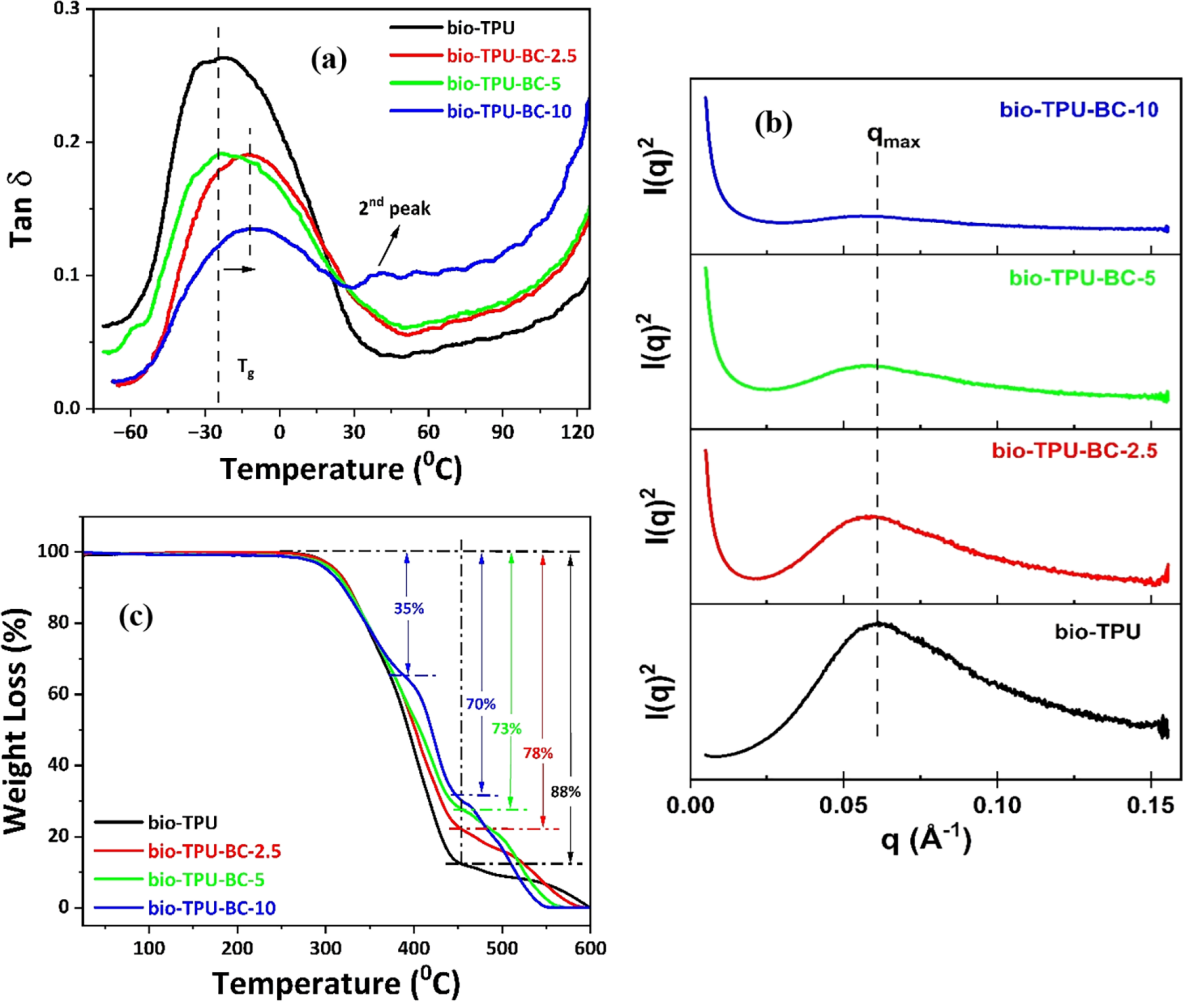
Plot of (a) tan δ vs temperature. (b) Shifting of peak maxima (*q*_max_) of the *I*(*q*)^2^ vs *q* plot to a lower *q* value, which suggests increased interdomain spacing and (a) TG weight loss vs temperature plot for bio-TPU/BBC biocomposites.

The raw 2D SAXS detector images of the bio-TPU and bio-TPU/BBC biocomposites mounted vertically are displayed in Figure S5a,d in the supporting information. The resultant Lorentz corrected scattering intensity from the SAXS measurements is plotted against the scattering wave vector in Figure 5b.

The lamellar structure of the bio-TPU results in a peak observed at *q* ∼0.06 Å^–1^ corresponding to interdomain spacing.^46,48^ After the incorporation of BBC, a clear displacement of the peak toward a lower *q* value, as well as broadening of the characteristic peak compared to bio-TPU, is evident from the Gaussian fitted peak maxima of the plot of *I*(*q*)^2^ vs *q*. Such shifting of the peak maxima leads to a substantial increase in interdomain spacing (*d*) from 11.22 to 12.09 nm, calculated from Gaussian peak fitting using Bragg’s Equation, listed in Table 5. It is important to mention that earlier investigations of TPU-based nanocomposites revealed that the interdomain distance of TPU was decreased upon the addition of fillers, demonstrating selective reinforcement in the HS of TPU associated with microphase separation.^46,48,53,60^ In contrast to that, our present findings on the increase of interdomain spacing demonstrate that BBC has hardly interacted with the HS of bio-TPU. Instead, favorable reinforcement of the SS of bio-TPU was achieved by preferential embedment within this phase.^48,53^ Such preferential embedment of BBC impacted SS crystallinity through nucleation and ultimately led to microphase separation at a higher loading (10 wt %) as observed earlier in DSC and DMA, respectively. From the Lorentz corrected data and peak maxima of the *I*(*q*)^2^ vs *q* plot, the long period (*L*_w_), crystalline lamella thickness (*L*_c_), amorphous lamella thickness (*L*_a_), and percentage of crystallinity (χ_c_) were calculated using the correlation function analysis according to eqs 1–3 and are listed in Table 5.

1 $$L_{w}=\frac{2\pi }{q_{\max}}$$

2 $$L_{w}=L_{c}+L_{a}$$

3 $$\chi_{c}=\frac{L_{c}}{L_{w}}$$

**Table 5:** Results of SAXS Study Parameters from Correlation Function Analysis

	interdomain spacing from peak fit	correlation function analysis			
sample	*d* (nm)	*L*_w_ (nm)	*L*_c_ (nm)	*L*_a_ (nm)	χ_c_ (%)
bio-TPU	11.22	9.5	2.7	6.8	29.3
bio-TPU-BC-2.5	11.60	10.1	2.8	7.3	28.2
bio-TPU-BC-5	11.73	9.5	3.1	6.4	33.2
bio-TPU-BC-10	12.09	8.8	3.5	5.3	39.6

It is noted that for bio-TPU-BC-2.5, *L*_w_ and *L*_a_ have slightly increased, while crystallinity has decreased compared to pristine bio-TPU. These results indicate that such a low BBC content leads to amorphous miscibility in the system without much interference with the crystalline arrangement of bio-TPU with the low crystallinity leading to maximum extensibility as inferred earlier in the mechanical property analysis.^61^ Beyond 2.5 wt % loading, *L*_a_ and *L*_w_ decreased as usual. Contrary to that, the highest percentage of crystallinity corresponding to bio-TPU-BC-10 leads to a maximum Young’s modulus. Meanwhile, a steady increase in *L*_c_ with BBC loading from 2.5 to 10 wt % indicates the increase in local crystallinity at the highest BBC loading.

The thermal stability of bio-TPU and bio-TPU/BBC biocomposites was further studied in a N_2_ atmosphere by TGA (Figure 5c). Bio-TPU-BC-10 has the best thermal degradation performance, with *T*_d‑50%_ at 420 °C and 70% weight loss at 450 °C. The degradation temperature at 50% weight loss (*T*_d‑50%_) was enhanced by the addition of BBC particles (Table 6). The preferential embedment of BBC in the SS phase resulted in the participation of a smaller amount of amorphous SS in the phase transformation, leading to improved thermal stability.^62^ These findings were compared with earlier reported literature using conventional carbonaceous and inorganic filler-based TPU systems. It has been observed that a 12 wt % loading of CB has led to a 36.32 °C improvement in maximum thermal decomposition temperature compared to neat TPU.^49^ In another study, an increase in residual mass percentage with an increase in CB loading at 600 °C from 3% (pure TPU) to 9% (TPU + 7 wt % CB composite) has been observed. By analogy, our findings on the gradual increase of residual mass percentage with an increase in BBC loading from 12% (for neat bio-TPU) to 30% (bio-TPU-BBC-10) at 450 °C showcase a logical agreement with previous conducted research.^50^ Additionally, TPU/fumed silica nanocomposites are reported to show a 3 °C increase in the onset degradation temperature, 5 °C increase in the maximum decomposition temperature, and 9 °C increase in final degradation temperature up to an optimal loading of 1 phr nanosilica content.^52^ However, it can be concluded that further work is needed to benchmark the performance of BBC as a sustainable filler against those of these more established systems.

**Table 6:** Results of TG Analysis of Bio-TPU/BBC Biocomposites

sample	*T*_d‑50%_ (°C)	weight loss at 450 °C (%)
bio-TPU	394	88
bio-TPU-BC-2.5	400	78
bio-TPU-BC-5	406	73
bio-TPU-BC-10	420	70

## Conclusions

A major challenge associated with the variable size of biochar from different sources was addressed in this study through a standardized ball-milling approach. Using this method, we successfully prepared sustainable biocomposites with enhanced mechanical and thermal properties by reinforcing bio-based TPU with ball-milled biochar (BBC), which preferentially embeds in the soft segments (SS) of the TPU matrix, as indicated by DSC, DMA, and SAXS analyses. Remarkably, even a low BBC loading of 2.5 wt % led to significant improvements in UTS and EB compared to neat bio-based TPU. Although 10 wt % biochar loading is generally recommended to boost Young’s modulus without sacrificing flexibility, our findings show that at 10 wt % BBC loading, maximum Young’s modulus (YM) and thermal stability were achieved, albeit at the cost of reduced UTS and EB. Furthermore, this strategy promotes environmental sustainability by reducing pollution, conserving energy, and preserving natural resources.

## Supplementary Material



## Notes

Characterization techniques, SEM, C-KLL Auger spectra, XPS composition report and Raman analysis and plots, BET plots, XRD pattern, and 2D SAXS detector images of bio-TPU/BBC biocomposites (PDF)
