# Supplementary material for: Microstructural Characterization of Ball-Milled Biochar and Its Reinforcing Efficiency in Biobased Thermoplastic Polyurethane through Preferential Embedment in the Soft Segment
Source: ACS Sustain Resour Manag. 2025 Sep 9;2(9):1719–30. doi: 10.1021/acssusresmgt.5c00225 (PMC12478856; doi:10.1021/acssusresmgt.5c00225)
Supplement: Supplementary file 1 [file rm5c00225_si_001.pdf]

# Supporting Information

## Microstructural Characterization of Ball-milled Biochar and its Reinforcing Efficiency in Biobased Thermoplastic Polyurethane through Preferential Embedment in the Soft-segment

Kunal Manna [a,*], Chaoying Wan [a], Jaipal Gupta [a], James J. C. Busfield [b], Biqiong Chen [c], and Ton Peijs[a,*]

[a] WMG, Centre for Polymers and Composites (CPC), University of Warwick, Coventry, CV4 7AL, UK

[b] School of Engineering and Materials Science, Queen Mary University of London, Mile End Road, London, E1 4NS, UK

[c] School of Mechanical and Aerospace Engineering, Queen's University Belfast, Stranmillis Road, Belfast, BT9 5AH, UK

Corresponding Author Email: Kunal.Manna@warwick.ac.uk, Ton.Peijs@warwick.ac.uk,

**Supporting Information -I (SI-I)**

## Characterization Techniques for Biochar

### *XRD*

X-ray diffraction (XRD) measurements were made in a reflection geometry using a Panalytical Empyrean equipped with a Cu $k_\alpha$ source in a Bragg-Brentano setup. Measurements were made between 7 and 70° $2\theta$ and a total collection time of 40 min. The samples were rotated during the measurement.

### *XPS*

The X-ray photoelectron spectroscopy (XPS) data were collected at the Photoemission RTP, University of Warwick. The samples investigated in this study were attached to electrically conductive carbon tape, mounted to a sample bar with a layer of filter paper between the sample and sample bar to ensure electrical isolation and hence mitigate differential charging, before being loaded into a Kratos Axis Ultra DLD spectrometer which possesses a base pressure below $1 \times 10^{-10}$ mbar. XPS measurements were performed in the main analysis chamber, with the sample being illuminated using a monochromated Al $K_\alpha$ x-ray source (h$\nu$ = 1486.7 eV). The measurements were conducted at room temperature and at a take-off angle of 90° with respect to the surface parallel. The core level spectra were recorded using a pass energy of 20 eV (resolution approx. 0.4 eV), from an analysis area of 300×700 microns. The work function and binding energy scale of the spectrometer were calibrated using the Fermi edge and $3d_{5/2}$ peak recorded from a polycrystalline Ag sample prior to the commencement of the experiments. To prevent surface charging, the surface was flooded with a beam of low energy electrons from a charge neutraliser throughout the experiment and this necessitated recalibration of the binding energy scale. To achieve this, the C-C/C-H component of the C 1s spectrum was referenced to 284.8 eV. The data were analysed in the CasaXPS package using Shirley backgrounds and mixed Gaussian-Lorentzian (Voigt) line shapes, except for the $sp^2$ C-C component where a LA (1.2,3,5) line shape was used. For compositional analysis, the analyser transmission function has been determined using clean metallic foils to determine the detection efficiency across the full binding energy range.

*RAMAN*

The graphitic structure of the ball milled and raw biochar were characterized using a Renishaw Invia [Gonzo] Raman spectrometer, at a wavelength of 532 nm. The laser was set at 5 mW and imaging was conducted under a 30 μm slit.

*SEM*

Biochar morphology and tensile fractured surface of bio-TPU-BC biocomposites were investigated through scanning electron microscopy (SEM) in a Tescan Clara scanning electron microscope at 5 kV, to check the physical appearance and determine the particle size of BBC. For SEM sample preparation, BBC powder was deposited on carbon tape and the tensile fractured surfaces of Bio TPU-BC biocomposites were pasted on the same and gold coated before analysis.

*BET*

The Brunauer-Emmett-Teller (BET) specific surface areas of RBC and BBC were obtained from the $N_2$ adsorption isotherms recorded at 77 K (Anton Paar Kaomi for Nova, St 4 on NOVA 800) at the relative pressure range from 0.03 to 1. In this method, the sample was first degassed for 12 h by increasing the temperature to 60 °C and applying vacuum. Later, the amount of adsorbed $N_2$ gas onto the surface of a known amount of sample is measured as a function of relative pressure. Finally, from the obtained isotherm, the amount of the required $N_2$ gas for covering the external and the accessible internal pore surfaces of the sample with a complete monolayer of $N_2$ gas was determined using the BET equation.

*Mechanical testing*

Tensile tests of bio-TPU and bio-TPU/BBC biocomposites were carried out using an AGS-X, Shimadzu tensile tester. Tests were carried out according to the ISO 5893 standard. A type 2 dumbbell test specimen was used, and at least 5 specimens were tested for each set. The tests were carried out at a cross-head speed of 500 mm/min using a 10 kN load cell with wedge grips.

*Hardness*

Shore D hardness test was carried out on samples of 6 mm thickness using a MonTech Hardness tester (Germany).

*SEM*

Biochar morphology and tensile fractured surface of bio-TPU-BC biocomposites were investigated through scanning electron microscopy (SEM) in a Tescan Clara scanning electron microscope at 5 kV, to check the physical appearance and particle size of the BBC. For SEM sample preparation, BBC powder was deposited on carbon tape and tensile fracture surfaces of bio-TPU-BC biocomposites were pasted on the same and gold-coated before analysis.

*TGA*

The thermal stability of all biocomposites with a sample weight ranging from 7 to 10 mg were investigated using a Mettler Toledo TGA instrument. All experiments were carried out under $N_2$ atmosphere and were heated from 30 to 600 °C at a heating rate of 10 °C/min in an alumina crucible, with a nitrogen flow of 50 mL/min.

*DSC*

Melting ($T_m$) and crystallization transitions ($T_c$) of bio-TPU and the bio-TPU-BC biocomposites were measured in a Mettler Toledo DSC 1 STARe system. All the samples, with weights ranging from 5 to 10 mg were used for scanning in the temperature range of −50 to 200 °C under a nitrogen flow of 50 mL/min. In the first cycle of the temperature scan, each sample was heated at a rate of 10 °C/min up to 200 °C and kept isothermally for 5 min to remove all the thermal history. Then the sample was cooled to −50 °C at 10 °C/min. The samples were reheated up to 200 °C at 10 °C/min. The crystallization temperature ($T_c$) was measured from the exothermic peak. Melting temperature ($T_m$) was determined from the endothermic peak of 2$^{nd}$ heating cycle.

*DMA*

The viscoelastic properties of the biocomposites were investigated using dynamic mechanical analysis (DMA). Sample specimens of dimension 10 × 4.5 × 1 mm (l × w × t) were cut out from sheets. The measurements were conducted in an air atmosphere using a TRITON

Tritec 2000 DMA instrument in tension mode in a temperature range from -70 to 150 °C, at a heating rate of 2 °C/min. The strain amplitude and frequency were 1% and 1 Hz, respectively.

*SAXS*

Small angle X-ray Scattering (SAXS) measurements were performed on a Xenocs Xeuss 2.0 equipped with a Cu $k_\alpha$ source collimated by two sets of scatter less slits. A Pilatus 300k detector mounted on a translation stage was used to record the scattered signal. Measurements were made with a sample-to-detector distance of 2.499 (5) m and a sample chamber filled with air. Giving a $q$ range for the detector between 0.005 $Å^{-1}$ and 0.156 $Å^{-1}$. The magnitude of the scattering vector ($q$) is given by $q = {}^{4\pi \sin\theta}/_{\lambda}$, where $2\theta$ is the angle between the incident and scattered X-rays and λ is the wavelength of the incident X-rays. A Pilatus 100k detector was also mounted at an angle of 36° to the X-ray beam and at a distance of 0.163 (2) m, giving a $2\theta$ range of 19 to 45°. For each sample, two pieces were cut and mounted such that the horizontal direction of the dog bone was known. One of these was mounted horizontally in the SAXS instrument and one vertically. Scattering data were then collected for 20 min. Azimuthal integrations of the 2D scattering data and background subtractions were performed using Xenocs XSACT software.[1] However, for data containing diffraction features, the measured intensity needs to be Lorentz corrected. The Lorentz correction factor is needed because the diffraction lines have a finite width, and the measured intensity is measured at a single orientation. This correction is typically used in single crystal and powder diffraction measurements and should only be used in SAXS experiments with diffraction peaks.[2,3]

*Correlation function analysis*

This analysis method focuses on the diffraction peak of a lamellar structure. The method is sensitive to oscillations in electron density, which for polymers occurs in lamellar structures within crystalline and amorphous regions.[4]

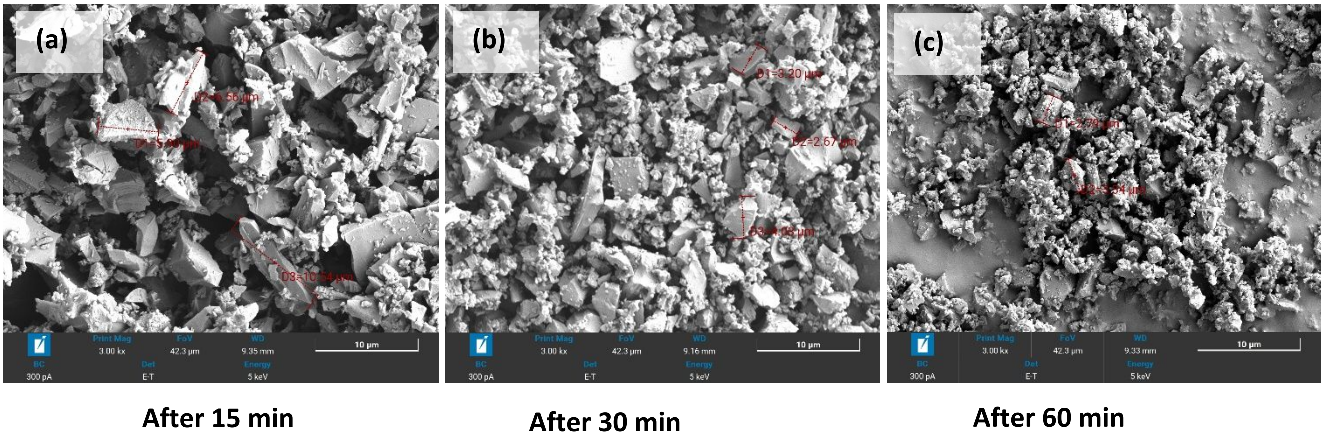


**Figure S1.** (a-c) SEM images of biochar after 15-, 30- and 60-min ball milling.

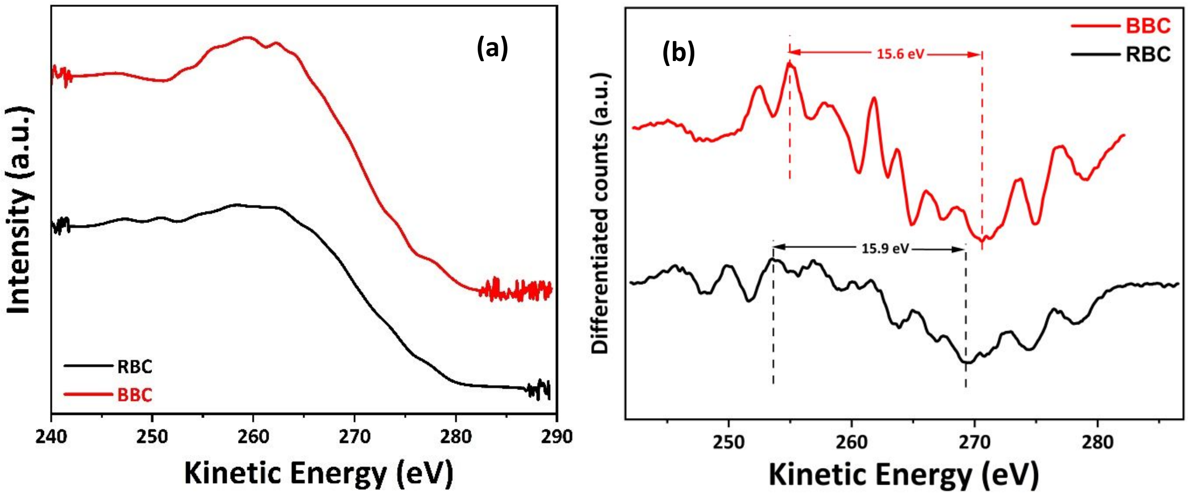


**Figure S2.** (a) X-ray induced C-*KLL* Auger spectra, (b) differentiated C KLL Auger plot for calculation of D-parameters, showing decrease in D-parameter corresponding to BBC.

**Table S1:** Relative percentage of the different bonding regions of RBC and BBC

| RBC | | | BBC | |
|---|---|---|---|---|
| | | | | |
| **C 1s / K2p region** | | | **C 1s / K2p region** | |
| *Binding energy (eV)* | *% of region* | *Bonding environment* | *Binding energy (eV)* | *% of region* |
| 284.39 | 53.3 | sp2 C-C | 284.27 | 33 |
| 290.8 | 2 | sp2 shake-up | 290.68 | 1.2 |
| 284.8 | 21.7 | sp3 C-C/C-H | 284.8 | 37.1 |
| 285.89 | 9.8 | C-O-C | 285.64 | 11.9 |
| 286.96 | 5.5 | C-OH | 286.75 | 7.6 |
| 288.1 | 2.9 | C=O | 287.88 | 1.9 |
| 289.36 | 3.6 | O=C-O | 289.04 | 6.7 |
| 293.19 | 0.6 | K2p3/2 | 293.17 | 0.3 |
| 295.91 | 0.5 | K2p1/2 | 295.89 | 0.3 |
| | | | | |
| **C KLL Auger region** | | | **C KLL Auger region** | |
| *d-parameter (eV)* | *sp2 %* | | *d-parameter (eV)* | *sp2 %* |
| 15.9 | 20 | | 15.6 | 15 |
| | | | | |
| **O 1s region** | | | | |
| *Binding energy (eV)* | *% of region* | *Bonding environment* | *Binding energy (eV)* | *% of region* |
| 531.13 | 23.7 | O=C | 530.9 | 12.4 |
| 532.49 | 44.4 | O-C | 532.12 | 49.9 |
| 533.84 | 28.7 | O*-(C=O) | 533.57 | 36.4 |
| 535.05 | 3.3 | H2O | 535.13 | 1.3 |
| | | | | |
| **N 1s region** | | | | |
| *Binding energy (eV)* | *% of region* | *Bonding environment* | *Binding energy (eV)* | *% of region* |
| 400.24 | 100 | Amine / amide | N/A | 0 |

## Supporting Information -II (SI-II)

*Raman Analysis*

The full Raman spectrum (200–3500 $cm^{-1}$) of BBC is shown in **Figure S3a** and the Raman spectra of RBC and BBC between 1000 to 1800 $cm^{-1}$ in **Figure S3b**, have almost overlapped each other without significant changes in either position or intensity of both the D (~1340 $cm^{-1}$) and G (~1590 $cm^{-1}$) bands. This implies that the defects or disorder induced (denoted by the D band) after ball milling are insignificant, keeping the $sp^2$ graphitic domain (denoted by the G band) intact, thereby confirming that ball milling is a non-destructive method

for inducing nanoscale changes in biochar microstructure. For further information, the Raman spectra of RBC and BBC were deconvoluted to give insight into their D, D′, D″ and G bands as depicted in **Figure S3c and d** respectively. The D′ band around ~1200 $cm^{-1}$ can be correlated to the $sp^3$ phase of amorphous carbons, while the D″ band around ~1550 $cm^{-1}$ is subject to disagreement and debate. Some researchers claim that it arises from different states within graphitic crystals and their associated phonon density while others have assigned it to vibrations of C–H groups on hydrogenated carbons. However, it is observed from **Figure S3c** that the D′ peak intensity has slightly increased in BBC while deconvoluted G peak intensity has slightly decreased compared to RBC indicating the presence of more $sp^3$ amorphous carbons in BBC after ball milling. This observation further strengthens our earlier findings of the $sp^2$ to $sp^3$ transition based on XPS analysis.

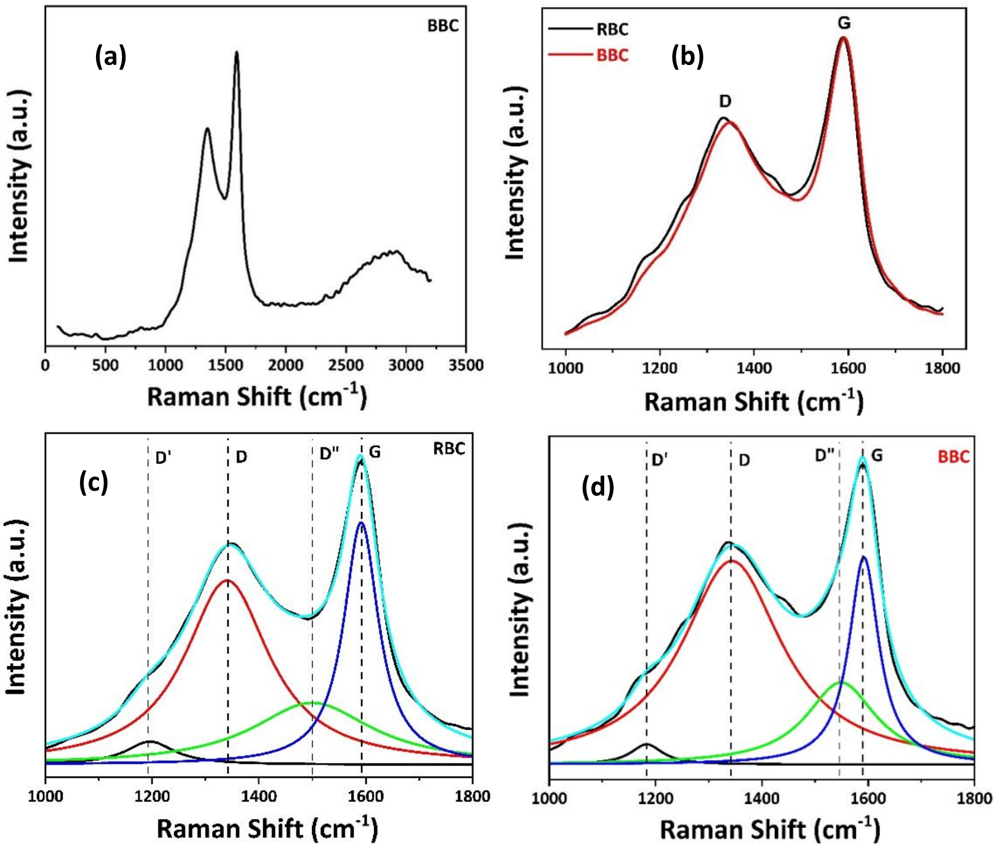


**Figure S3.** (a) Full Raman spectrum of BBC. (b) Raman spectra of RBC and BBC. Deconvoluted Raman spectra of (c) RBC and (d) BBC, showing increased D′ peak intensity and decrease in deconvoluted G peak intensity in BBC.

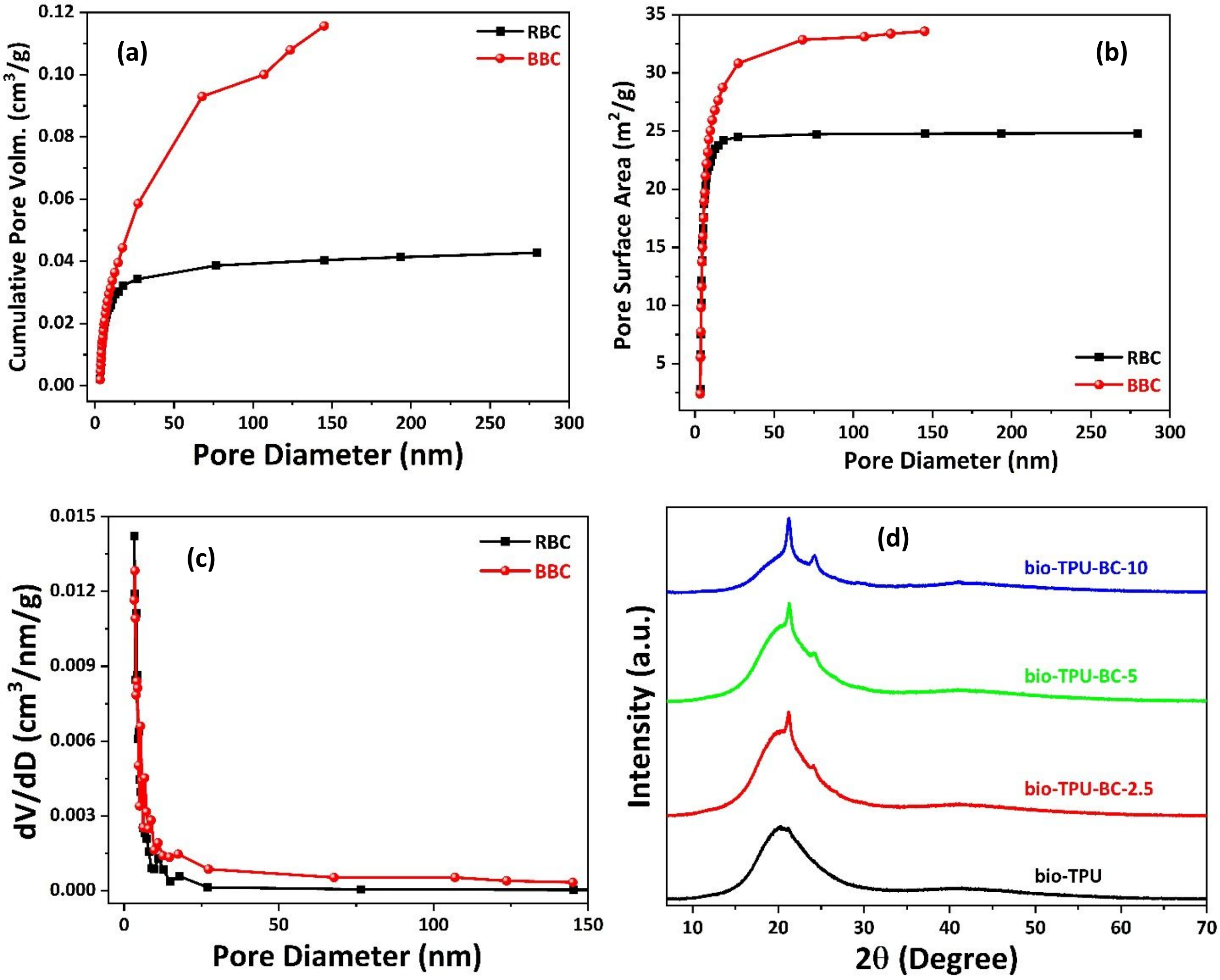


**Figure S4.** (a) Plot of (a) cumulative pore volume vs pore diameter, (b) pore surface area vs pore diameter. (c) Pore size distribution curves of RBC and BBC. And Powder XRD pattern of bio-TPU/BBC biocomposites.

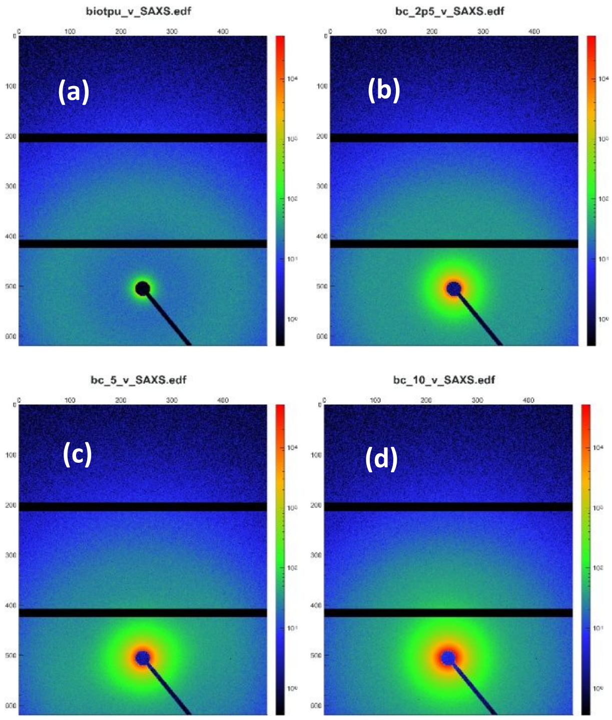


**Figure S5.** 2D SAXS images of (a) bio-TPU, (b) bio-TPU-BC-2.5, (c) bio-TPU-BC-5, (d) bio-TPU-BC-10.

The bio-TPU (Figure S5a) showed a clear isotropic (all in-plane orientations) scattering ring from the lamellar structure. With increased BBC loading, the scattering at low angles increased due to the BBC particle.

**References:**

1. Xenocs, "XSACT: X-ray Scattering Analysis and Calculation Tool." xsact.xenocs.com, 2023. SAXS & WAXS data analysis software — Version 2.10.

2. Xenocs, "XSACT: X-ray Scattering Analysis and Calculation Tool." xsact.xenocs.com, 2023. SAXS & WAXS data analysis software — Version 2.10. For data measured on the SAXS instrument at Warwick this software package is generally used to make this correction. Included in the data folder for this experiment is a .pdf describing the correction utility used for the Lorentz correction.

3. Cser, F. (2001), About the Lorentz correction used in the interpretation of small angle X-ray scattering data of semicrystalline polymers. J. Appl. Polym. Sci., 80: 2300-2308

4. *http://www.sasview.org/* - The SasView package has a range of SAXS data fitting methods including a module for correlation function analysis, used for the analysis here. The help page for this is available here: https://www.sasview.org/docs/user/qtgui/Perspectives/Corfunc/corfunc_help.html
